# Ag-Modified ZnO for Degradation of Oxytetracycline Antibiotic and Reactive Red Azo Dye

**DOI:** 10.3390/antibiotics11111590

**Published:** 2022-11-10

**Authors:** Khemika Wannakan, Kamonpan Khansamrit, Teeradech Senasu, Tammanoon Chankhanittha, Suwat Nanan

**Affiliations:** Materials Chemistry Research Center, Department of Chemistry and Center of Excellence for Innovation in Chemistry (PERCH-CIC), Faculty of Science, Khon Kaen University, Khon Kaen 40002, Thailand

**Keywords:** Ag-decorated ZnO, photodegradation, reactive red dye, oxytetracycline, sunlight

## Abstract

It is known that low electron-hole separation efficiency is the major disadvantage influencing low photoactivity of the UV-active ZnO photocatalyst. To solve this drawback, the excellent fabrication technique has been used to disperse silver metal on ZnO surface. In this study, an addition of silver content up to 15 wt% was carried out. The 5Ag-ZnO sample, comprising 5 wt% of silver metal, displayed a hexagonal wurtzite structure, and a band gap of 3.00 eV, with high sunlight-active photocatalytic performance of 99–100% and low photo-corrosion problem. The complete degradation of oxytetracycline (OTC) antibiotic and reactive red dye 141 (RR141) dye under natural sunlight was achieved. The highest rate constant of 0.061 min^−1^ was detected. The enhancement of the performance is mainly due to lowering of the electron-hole recombination rate. Dispersion of silver on ZnO causes the generation of the Schottky barrier at the interface between Ag and ZnO, so that improvement of quantum efficiency and enhancement of the resultant photoactivity could be expected. Furthermore, good distribution of metallic silver also causes a red shift in absorption of light toward the visible spectrum. This is strongly attributed to the surface plasmon resonance effect, which occurred after successful decoration of the noble metal on ZnO. The photocatalyst, with great structural stability, still maintains high photocatalytic efficiency even after five times of use, implying its excellent cycling ability. The present finding offers a new road to generate a silver decorated ZnO photocatalyst for the complete removal of dye and antibiotics contaminated in the environment.

## 1. Introduction

It has been reported that toxic dyes have been detected in wastewater effluents from numerous factories, including textile and pharmaceutical industries [1,2,3,4]. Reactive dyes, comprising azo chromophores, are toxic and mutagenic to living organisms. Even a low concentration of the azo dye in water is visible and undesirable [4,5,6]. Therefore, searching for a cost-effective technology for the treatment of water containing this toxic dye is needed [7,8,9,10,11]. Oxytetracycline (OTC) is a typical drug used for the treatment of bacterial-infectious diseases [12,13]. However, this antibiotic causes a serious problem to the environment. After OTC is metabolized in the body, the toxic byproducts could enter the natural water, causing the damage to the ecosystem and human health. Thus, degradation of the organic pollutants from the natural water is urgently required [14].

It is reported that various techniques have been applied for the removal of toxic contaminants in wastewater. However, major disadvantages in comparing the incomplete removal of the contaminants and the generation of secondary harmful pollutants are detected [15,16,17]. Alternatively, to reach the sustainable development goals, photocatalytic treatment has been used for the complete degradation of the organic contaminants [18,19,20,21]. Basically, the commercially available TiO_2_ photocatalyst has been used extensively. However, this photocatalyst is active only under UV light (5% of natural sunlight) [22,23,24]. Interestingly, the search for new photocatalysts with high performance under an economical solar energy is a promising research direction [9,10,11,12,13,14,19].

The ZnO photocatalyst has gained much attention because it shows excellent transport property, low cost of production, and versatile morphology [22,23,24,25,26]. Nevertheless, low sunlight performance and occurrence of the photo-corrosion are the main drawbacks influencing the practical application of ZnO [27]. According to the literatures, ZnO was synthesized by various routes [28,29,30,31,32,33,34,35]. Among these techniques, a chemical precipitation method has been used to prepare the semiconducting nanomaterials, which have the benefits of being inexpensive, simple, and easy to control [2].

In principle, the UV-active ZnO photocatalyst faces the problem of low performance due to low electron-hole separation efficiency. Both doping/decorating of the metal or creating a heterostructure has been used to improve the performance of the bare ZnO [9,18,19,20,25,26,27,33,34,35]. Interestingly, after well dispersion of the selected noble metals on ZnO, an increase of the visible light absorption is expected. The result is attributed to the effect of surface plasmon resonance (SPR) from the noble metal. In addition, an increase in photo-generated charge lifetime is also obtained. This will end up with the enhanced photocatalytic performance found in the metal decorated ZnO, compared to the pristine ZnO [9].

Synthesis of various metal-decorated semiconductors has been demonstrated [27,28,29,30,31,32,33,34,35,36]. The Ag/ZnO photocatalyst provides an enhanced photocatalytic performance compared to the bare ZnO, due to a dramatic increase of charge carries separation rate at the interface [9,28,29,30,31,32,33,34,35,36,37,38,39,40,41,42,43,44]. Silver/ZnO photocatalysts have been prepared by using numerous methods [45,46,47,48,49,50,51,52,53,54]. Among these techniques, the photocatalytic deposition is an alternative route for well dispersion of metallic nanoparticles on the semiconductor [45]. Ideally, this method consists of the photo-irradiation of the solution, which contains the bare semiconductor and metal source (metal salt) under controlled conditions. During the photo-deposition procedure, the excitation of the bare semiconductor gives rise to the formation of electron-hole pairs, which can reduce the metal ion. Furthermore, the photogenerated holes found in the valence band carry out the oxidation of sacrificial electron donors.

In the present research, the Ag-ZnO photocatalysts, with various silver loading, were fabricated. The bare ZnO was prepared first by a facile chemical precipitation method. After that the Ag-modified ZnO was then synthesized by using a photoreduction technique. This is more practical in terms of large-scale production of the catalyst, compared to our previous work based on hydrothermal synthesis of ZnO [9]. The hexagonal 5Ag-ZnO, with E_g_ of 3.00 eV, showed the lowest PL intensity, indicating the greatest photodegradation efficiency toward the removal of OTC and RR141 due to the improvement of electron-hole separation efficiency after well dispersion of silver metal on ZnO surface. The degradation of the dye and the antibiotic follows the first-order kinetic model. The silver-ZnO catalyst, with great chemical stability, retains promising photoactivity after the fifth time of use, indicating the excellent cycling ability of the sample. This work demonstrates how to create a promising photocatalyst for environmental remediation by uniform dispersion of silver metal on ZnO.

## 2. Experiment

### 2.1. Chemicals

All analytical grade chemicals were used. No further purification was carried out. The deionized water (DI, 18.2 MΩ.cm) was used throughout the experiment.

### 2.2. Preparation of the Photocatalysts

#### 2.2.1. Preparation of ZnO

A ZnO photocatalyst was synthesized using a chemical precipitation method [2]. In a typical procedure, about 2.1949 g of Zn(CH_3_CO_2_)_2_·2H_2_O was dissolved in 50 cm^3^ of DI water. Meanwhile, about 0.4000 g of NaOH were dissolved in 25 cm^3^ of DI water. The sodium hydroxide solution was added into the zinc acetate solution with continuous stirring. It was left at 100 °C for 6 h. The white solid was filtered, washed, and dried at 80 °C for 15 h before use.

#### 2.2.2. Preparation of Silver-ZnO

The well dispersion of silver metal on ZnO was carried out using a photoreduction route as follows [9,45]. About 0.3000 g of ZnO was well dispersed in 100 cm^3^ of DI water and then the AgNO_3_ solution was added. The mixture was stirred for 30 min, ensuring that the Ag^+^ ions can be adsorbed sufficiently on the ZnO surface. After that, the reaction mixture was illuminated under a Mercury lamp (125 W) for 1 h. The product was then filtered and washed. In the last step the sample was dried at 80 °C for 8 h before use. The preparation of silver-ZnO with 0, 5, 10, and 15 wt% of silver is known as ZnO, 5Ag-ZnO, 10Ag-ZnO, and 15Ag-ZnO, respectively.

### 2.3. Characterization

All samples were characterized according to the previous reports [2,9,12,13]. The chemical structure was determined by a powder X-ray diffraction method using a monochromatic Cu Kα radiation with a scanning rate of 0.02 degree per second. The FT-IR spectrum was recorded using a FT-IR spectrophotometer. Preparation of the sample was carried out using KBr pellets method. The morphology and the elemental composition of the prepared photocatalysts were investigated by scanning electron microscopy, transmission electron microscopy, energy dispersive X-ray spectroscopy, and elemental mapping. The UV-vis diffuse reflectance spectrum was investigated. The photoluminescence spectrum (PL) was elucidated.

### 2.4. Photocatalytic Study

The photocatalytic degradation of both OTC antibiotic and RR141 dye was studied under UV light (a Mercury lamp, 125 W, wavelength of 200–400 nm) and natural solar light [9,12]. In practice, the photocatalytic study was carried out in a pollutant solution of 10 ppm (total volume of 200 cm^3^, and photocatalyst loading of 50 mg). About 5 cm^3^ of the sample was collected at the given time [1,2,3,4,5]. The remaining concentration of OTC and RR141 was determined based on the measurement of absorbance at λ_max_ of 353 and 544 nm, respectively, using a UV-vis spectrophotometer.

The photoactivity toward degradation of the contaminant was calculated as follows.

Photoactivity (%) = (1 − C/C_0_) × 100% (1)

where C_0_ and C represents the initial concentration and the concentration at a given illumination time, respectively.

The photocatalytic performance of the prepared photocatalyst was also calculated from the rate of the photocatalytic degradation reaction, as follows:

dC/dt = −k_1_C (2)

ln(C_0_/C) = k_1_t (3)

where k_1_ is the first-order rate constant.

By examining the removal of OTC and RR141, the influence of some factors such as the initial contaminant concentration, solution pH, and catalyst loading on photoactivity was studied as well [2,9,12].

To investigate the main species involved in the removal of the toxic pollutants [6,7,8,9,10], incorporation of different scavengers including isopropyl alcohol (IPA), NaN_3_, EDTA-2Na and K_2_Cr_2_O_7_ were added to quench the hydroxyl radicals, superoxide anion radicals, holes, and electrons, respectively. Furthermore, KI was also incorporated as a scavenger of surface hydroxyl radicals and holes. Each quencher was added in the presence of the photocatalyst (50 mg).

To monitor the hydroxyl radicals (OH·), a dispersion of the prepared photocatalyst in terephthalic acid solution (TA) was carried out [9,12,13]. The creation of the radicals was monitored by using a spectrofluorometric method (λ_excitation_ of 315 nm).

The reusability of the photocatalyst was investigated by determining the photodegradation of both OTC antibiotic and RR141 dye for five cycles [12,13].

## 3. Results and Discussion

### 3.1. Characterization of the Prepared Photocatalyst

**Figure 1:**
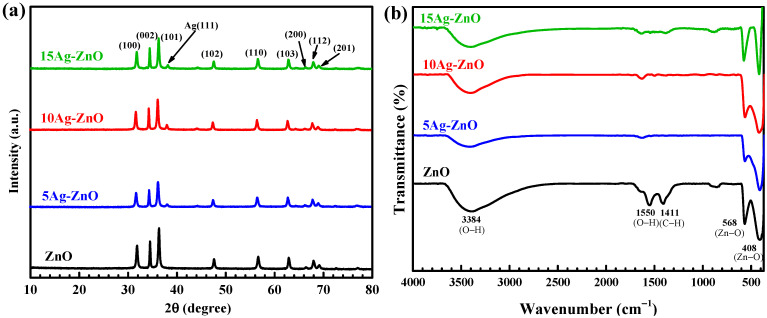
XRD patterns (**a**) and FT-IR spectra (**b**) of ZnO, 5Ag-ZnO, 10Ag-ZnO, and 15Ag-ZnO photocatalysts.

The chemical structure of the prepared photocatalyst was examined via X-ray diffraction method (Figure 1a,b). The pattern from the XRD diffractogram of the bare ZnO belongs to the hexagonal wurtzite structure (JCPDS No. 36-1451) [55,56]. The diffraction located at 2θ of 31.76°, 34.41°, 36.25°, 47.53°, 56.59°, 62.85°, 66.37°, 67.94°, and 69.08° corresponded to the reflection from crystal plans of the (100), (002), (101), (102), (110), (103), (200), (112), and (201), respectively. In the case of silver-ZnO, a new diffraction peak at about 38.25° was attributed to the reflection from the (111) crystal plane of metallic silver (face-centered cubic, FCC phase) according to the file (JCPDS No. 01-087-0718) [9,55]. The result confirms the successful creation of silver decorated ZnO. No diffraction signals were found. Neither Zn(OH)_2_ nor Ag_2_O were detected in the Ag-ZnO. The crystallite size of each sample was calculated using the Scherrer equation [9]. Sizes of about 25.97, 20.67, 20.97, and 21.39 nm were detected for the as-synthesized ZnO, 5Ag-ZnO, 10Ag-ZnO, and 15Ag-ZnO, respectively.

The functional groups found in the prepared photocatalyst were examined by a FT-IR spectroscopy. As seen from the FT-IR spectra in Figure 1b, the peaks located at 3384 and 1550 cm^−1^ were due to O-H stretching and bending modes from the adsorbed molecular water or hydroxyl group on ZnO surface, respectively [9]. The vibrational peaks at 408 cm^−1^ and 568 cm^−1^ were due to the stretching vibration from the Zn-O bond [9]. A slight decrease of the peak intensity with an addition of silver was observed. This is attributed to the existence of the Ag-Ag interaction from metallic Ag after well dispersion of the metal on ZnO [55].

**Figure 2:**
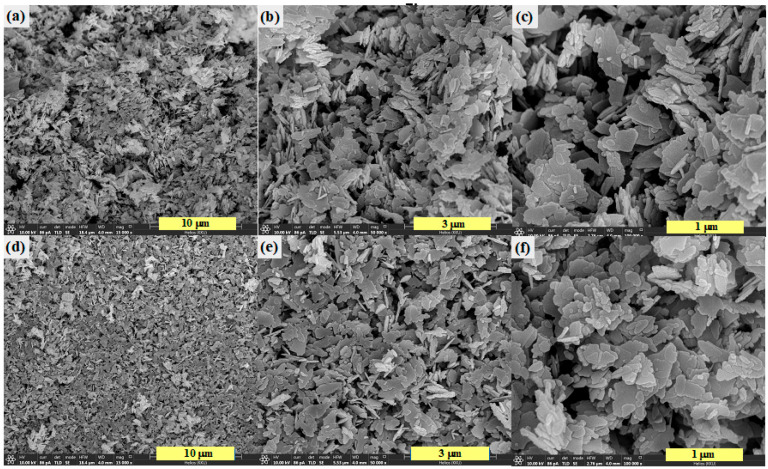
FE-SEM micrographs of bare ZnO (**a**–**c**), and 5Ag-ZnO photocatalyst (**d**–**f**).

The morphology of both ZnO and 5Ag-ZnO was elucidated by Scanning Electron microscopic (SEM) method. The SEM images of the pristine ZnO (Figure 2a–c) showed a thin plate-like morphological structure of about 0.30 µm × 0.50 µm. Meanwhile, The SEM images of the 5Ag-ZnO photocatalyst (Figure 2d–f) showed nearly the same morphology. As seen, the final morphology did not alter with the incorporation of silver. Thus, the presence of silver can be further confirmed using other techniques such as EDS-FESEM, TEM, and XPS.

**Figure 3:**
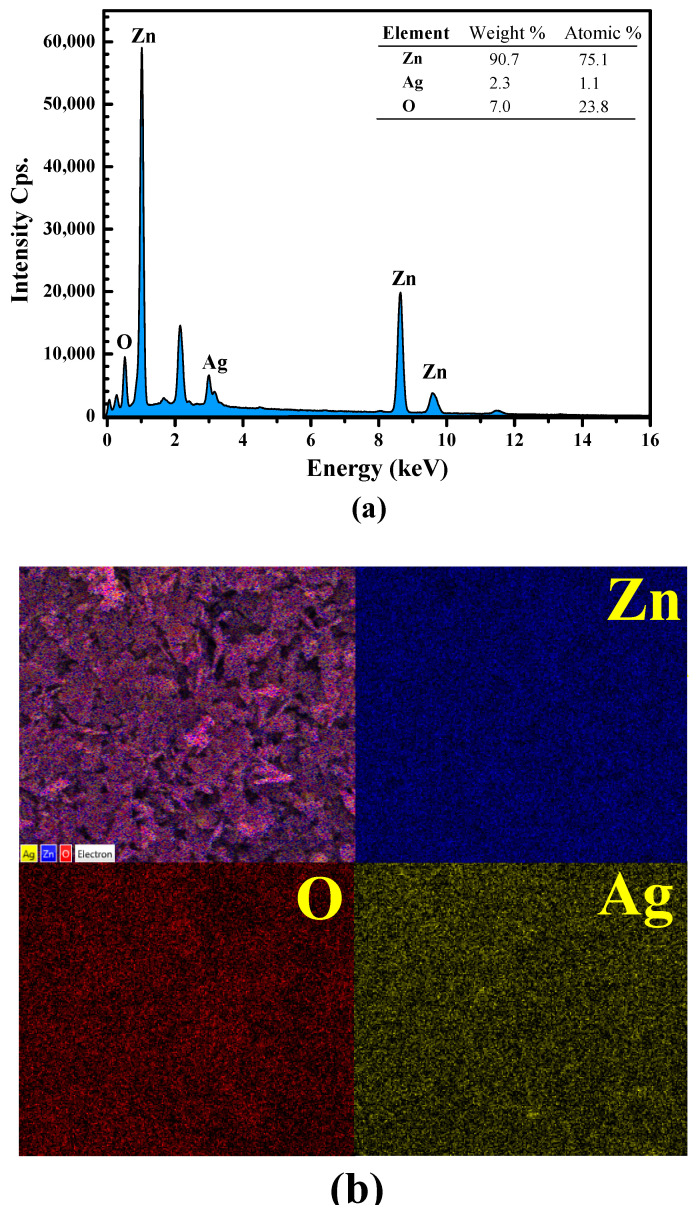
EDX spectrum (**a**), SEM image of the mapping area, and EDX elementary mapping of Zn Ag, and O elements (**b**) obtained from the 5Ag-ZnO photocatalyst.

Energy-dispersive X-ray spectroscopy (EDS) was used to determine the elemental compositions in the sample. Three elements, comprising Zn, O, and Ag, were detected in the synthesized silver decorated ZnO sample (Figure 3a). The atomic percentages of 75.1%, 1.1%, and 23.8%, were detected for Zn, Ag, and O, respectively. The result confirms the element composition in the prepared photocatalyst. Furthermore, the dispersion of all elements was elucidated by EDS elemental mapping (Figure 3b). The uniform distribution of Zn, O, and Ag elements was confirmed.

**Figure 4:**
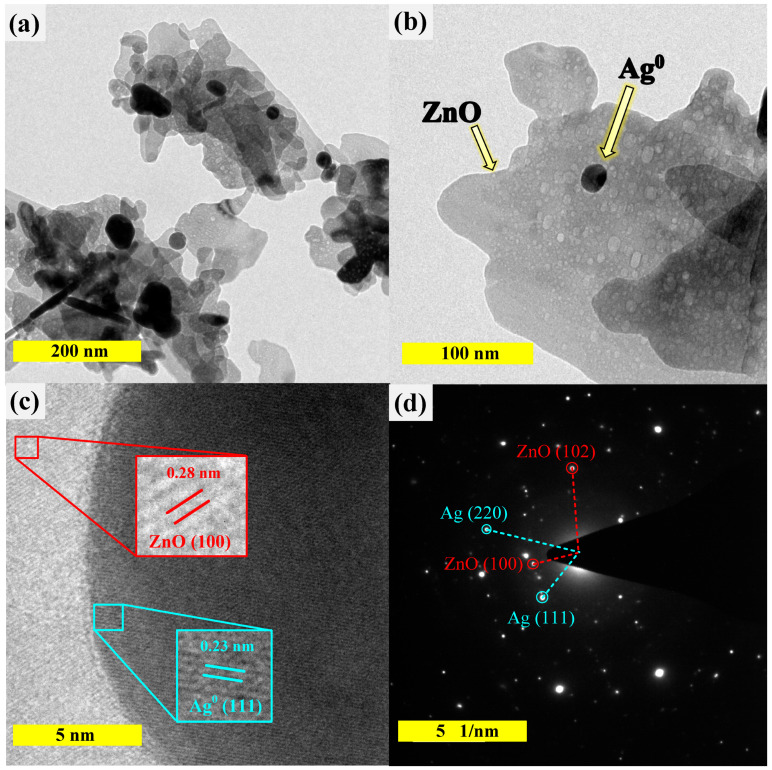
TEM images (**a**,**b**), HRTEM image (**c**), and SAED pattern (**d**) obtained from the 5Ag-ZnO photocatalyst.

The successful creation of silver-ZnO was also examined by the Transmission Electron microscopic (TEM) method. The TEM image of 5Ag-ZnO (Figure 4a) displayed a plate-like morphology of about 200 nm. The high magnification TEM image in Figure 4b showed the presence of spherical silver particles of 15–25 nm on the ZnO surface. The high-resolution TEM (HR-TEM) micrograph in Figure 4c showed the interplanar spacing of 0.23 nm and 0.28 nm. These are due to the existence of the (111) crystal plane of face-center cubic (FCC) silver metal and the (100) plane of ZnO, respectively. The SAED pattern shown in Figure 4d revealed the monocrystalline nature of the prepared photocatalyst [9]. The results agree well with those from XRD, FT-IR, and EDS-FESEM presented previously. Thus, the successful synthesis of Ag/ZnO heterojunction, via well dispersion of silver metal on ZnO surface, was demonstrated.

**Figure 5:**
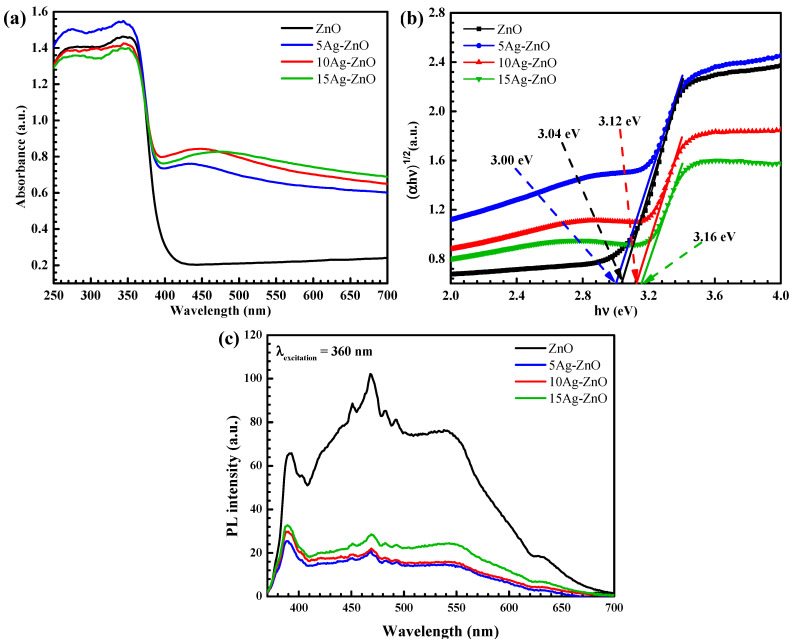
Diffused reflectance spectra (**a**), Tauc plot for determination of band gap (**b**), and PL spectra of all the prepared photocatalysts using λ_excitation_ of 360 nm (**c**).

The optical properties of the prepared photocatalyst were recorded using the UV-vis diffuse reflectance spectroscopic (DRS) method. The spectra in Figure 5a showed an absorption band edge of over 400 nm. The band gap (E_g_) was determined by the Kubelka–Munk equation [57]. The bare ZnO, direct-allowed transition semiconductor, provided a strong UV-light response comprising the absorption edge of 408 nm (E_g_ = 3.04 eV). It is clearly seen that an extension of the visible light response was observed with the incorporation of silver metal due to the SPR effect [9,55]. The 5Ag-ZnO sample provided an energy gap of 3.00 eV (absorption band edge of 413 nm). An improvement of charge carrier generation efficiency can be obtained with an increase in the light absorption over the UV-visible region, so that a remarkable enhancement of the resultant photoactivity could be reached [9,55].

To roughly compare the electron-hole recombination rate in the prepared photocatalysts, the photoluminescence (PL) spectroscopic method was performed [57]. According to the PL spectra in Figure 5c, the 5Ag-ZnO provided the lowest intensity in the PL spectra, implying the lowest electron-hole recombination rate in this sample. Therefore, the highest photocatalytic performance, compared to other samples, could be observed.

**Figure 6:**
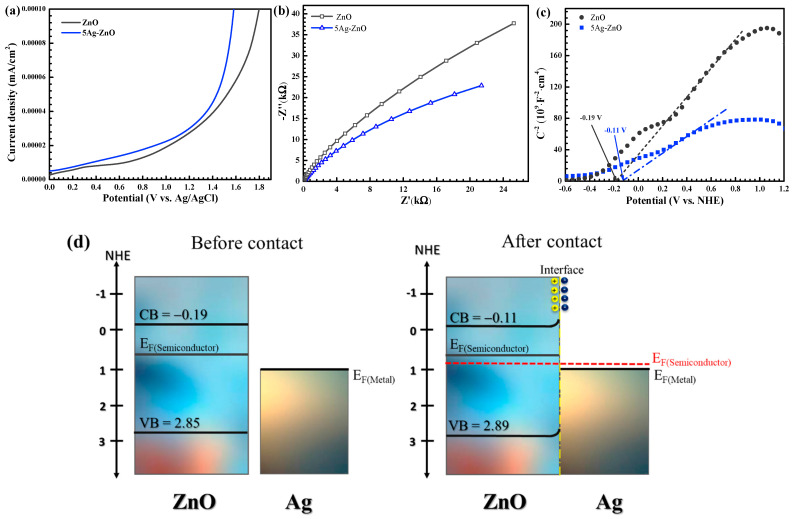
Linear sweep voltammetry (LSV) scans plots (**a**), electrochemical impedance spectroscopy (EIS) Nyquist plots (**b**), Mott–Schottky plots (**c**), and energy band structure of the 5Ag-ZnO photocatalyst both before and after contact (**d**).

The electrochemical technique was examined to find the spatial transfer and separation of charge carriers using the linear sweep voltammetry (LSV) and electrochemical impedance spectroscopic (EIS) methods [12,13]. The current density of 5Ag-ZnO was higher than that of the bare ZnO (Figure 6a). Therefore, it can be concluded that well dispersion of silver metal (Ag^0^) on ZnO provides an improvement of the charge separation efficiency, causing an increase in the resultant photoactivity [12]. The result agrees well with the trend of relative intensity found in the PL spectra.

The charge recombination rate was estimated by performing the EIS under light irradiation. In principle, the arc radius provides the information concerning the charge transfer process at electrode/electrolyte interface. A higher radius implies a greater charge-transfer resistance [12,13]. The 5Ag-ZnO displayed lower arc radius compared to the pristine ZnO (Figure 6b). This indicates the lowering of the resistance after well dispersion of silver on ZnO [9]. The 5Ag-ZnO with low resistance (low arc radius) is expected to gain great photocatalytic performance, compared to the bare ZnO. The results from PL, the LSV, and the EIS experiments strongly confirm the lowering of electron-hole recombination rate after well dispersion of silver on ZnO semiconductor. This causes a dramatic improvement to the resulting photoactivity.

To understand the mechanism regarding the enhance efficiency of the Ag-ZnO, the Mott–Schottky plot was elucidated for determination of the band structures of ZnO and silver-ZnO (Figure 6c). The details have been shown previously [9]. The flat band (V_FB_) level of the bare ZnO was found to be −0.19 eV while that of the 5Ag-ZnO was about −0.11 eV. The values are nearly the same as the conduction band (V_CB_) levels of the photocatalysts [12] so that the V_CB_ values of ZnO and silver-ZnO were found to be −0.19 and −0.11 eV, respectively. The prepared ZnO (E_g_ of 3.04 eV) showed a V_VB_ level of 3.23 eV. The silver-ZnO (E_g_ of 3.00 eV) displayed a V_VB_ value of 3.11 eV. In summary, the values of V_CB_ and V_VB_ levels obtained from ZnO (before generation of metal/semiconductor junction) and silver-ZnO (after generation of metal/semiconductor junction) were depicted in Figure 6d.

### 3.2. Photocatalytic Degradation of OTC Antibiotic and RR141 Dye

Photocatalytic performances of all the prepared catalysts were investigated by studying the photocatalytic degradation of OTC antibiotic and RR141 dye as two model organic contaminants.

#### 3.2.1. Photocatalytic Degradation of the Pollutants under UV Light

**Figure 7:**
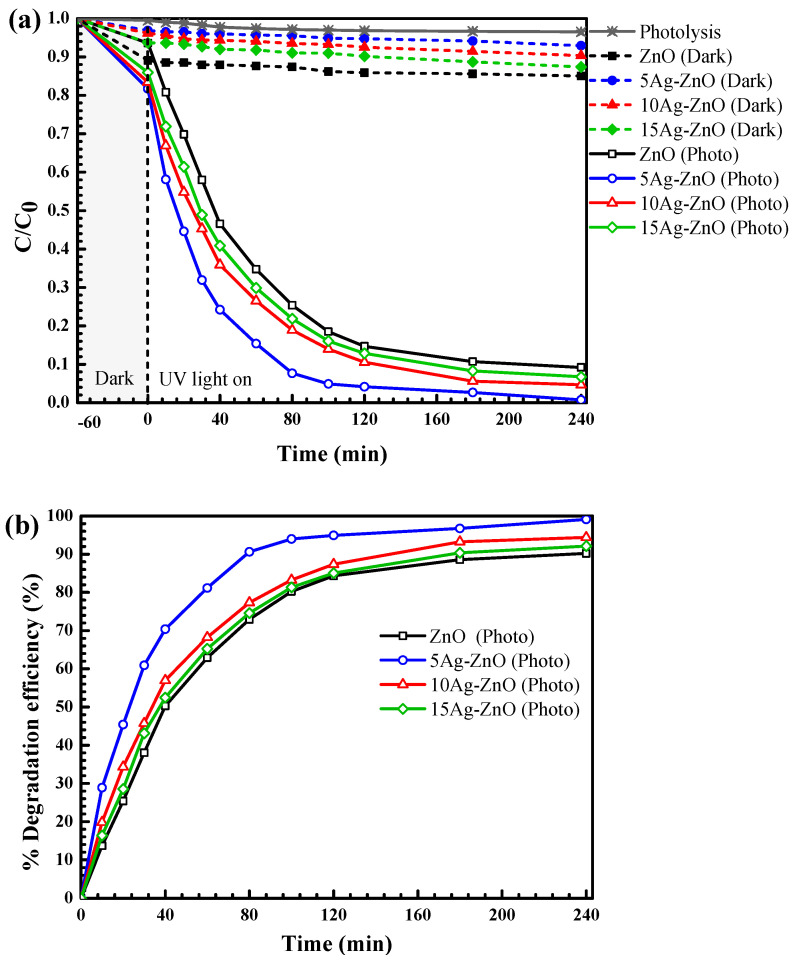
Plots of OTC antibiotic concentration vs. time (**a**) and the photodegradation efficiency (**b**) under UV light irradiation.

As seen in Figure 7a, the decreasing OTC concentration with irradiation time confirms the degradation of the antibiotic. The photolysis of OTC can be excluded. Less than 7% removal of OTC via adsorption process was detected in the presence of the 5Ag-ZnO photocatalyst. Interestingly, the enhanced degradation of OTC was observed under photo illumination. The performance reached 99% after 240 min (Figure 7b). The photoactivity of the 5Ag-ZnO is about 1.2 times higher than that of the bare ZnO. The increasing weight percentage of silver to 15 wt% somewhat results in lowering the performance. This may be due to aggregation of high silver content on the ZnO surface. Moreover, the addition of excess silver loading might end up with creation of the recombination center, which in turn results in lowering the photocatalytic performance.

**Figure 8:**
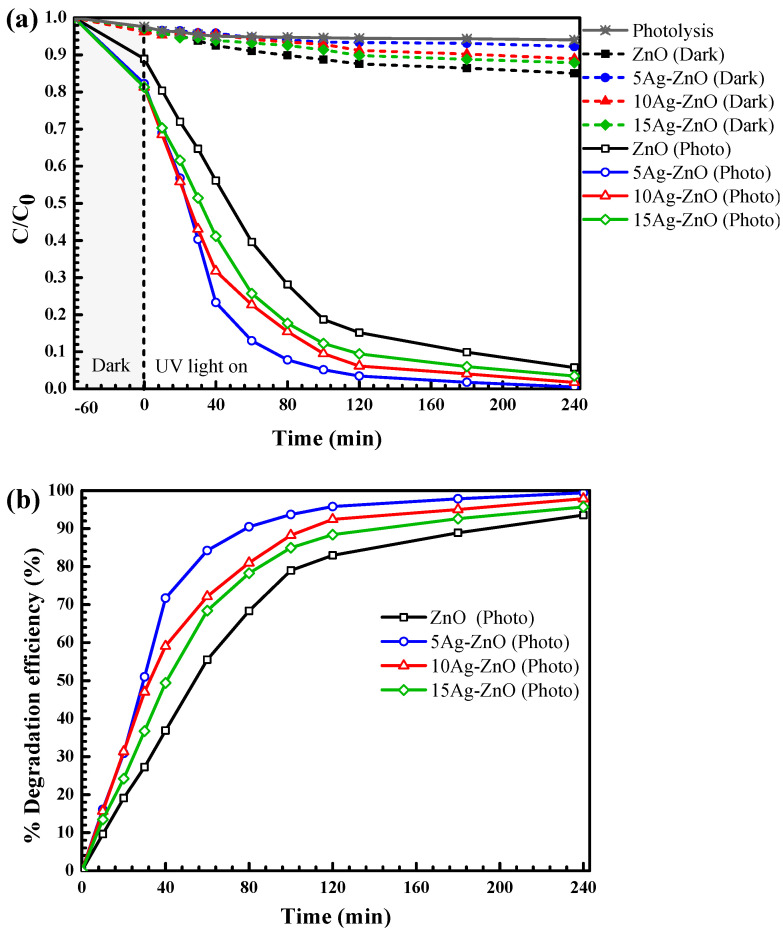
Plots of RR141 dye concentration vs. time (**a**) and the photodegradation efficiency (**b**) under UV light irradiation.

**Figure 9:**
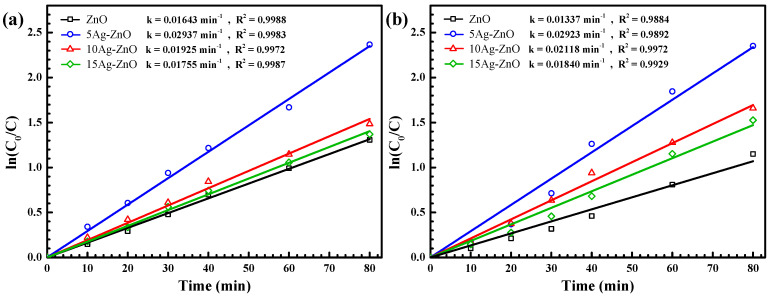
Plots of ln(C_0_/C) vs. time obtained from photodegradation of OTC antibiotic (**a**) and RR141 dye (**b**) under UV light irradiation.

When examining the RR141 degradation, Figure 8a shows a decrease of dye concentration over time. The photolysis of RR14 is negligible. In addition, lower than 8% of RR141 was adsorbed after incorporation of 5Ag-ZnO (in the dark). The enhanced photocatalytic performance of 99% was obtained after 240 min of photo irradiation (Figure 9b). The performance of the 5Ag-ZnO is about 1.1 times greater than that of the bare ZnO after 240 min.

**Figure 10:**
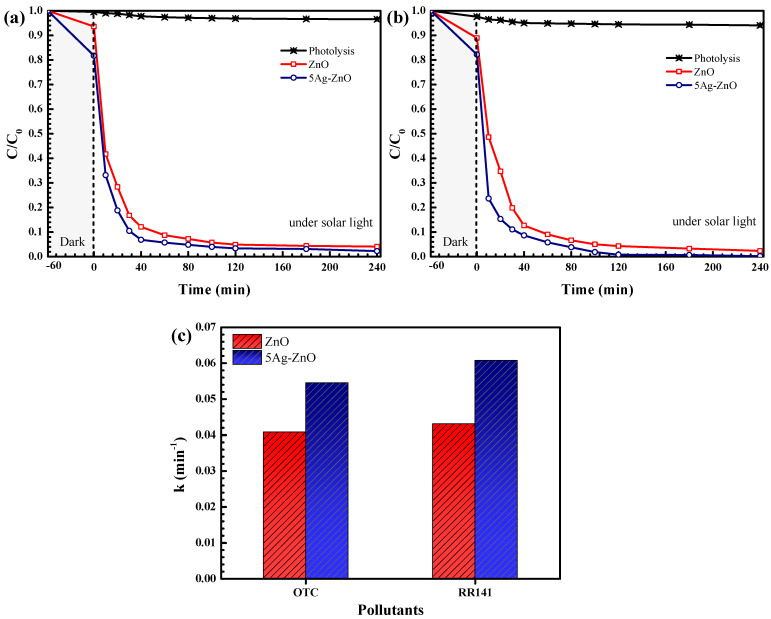
Lowering of OTC antibiotic (**a**) and RR141 dye (**b**) concentration with time under natural sunlight, and the rate constants of the photocatalysts toward degradation of OTC antibiotic and RR141 dye (**c**).

The photocatalytic degradation of OTC antibiotic and RR141 dye correlates well with the first-order reaction (Figure 10). The corresponding rate constants (k) of 0.0294 and 0.0292 min^−1^ were observed. The 5Ag-ZnO provides the highest rate constants among all prepared photocatalysts. This agrees well with the maximum photoactivity found in the previously presented silver decorated ZnO. All in all, the greatest photoactivity and the highest rate constant obtained from the 5Ag-ZnO photocatalyst is mainly due to the effective electron-hole separation efficiency after the successful construction of the silver decorated ZnO [9,55].

#### 3.2.2. Photodegradation of the Pollutants under Natural Sunlight

**Figure 11:**
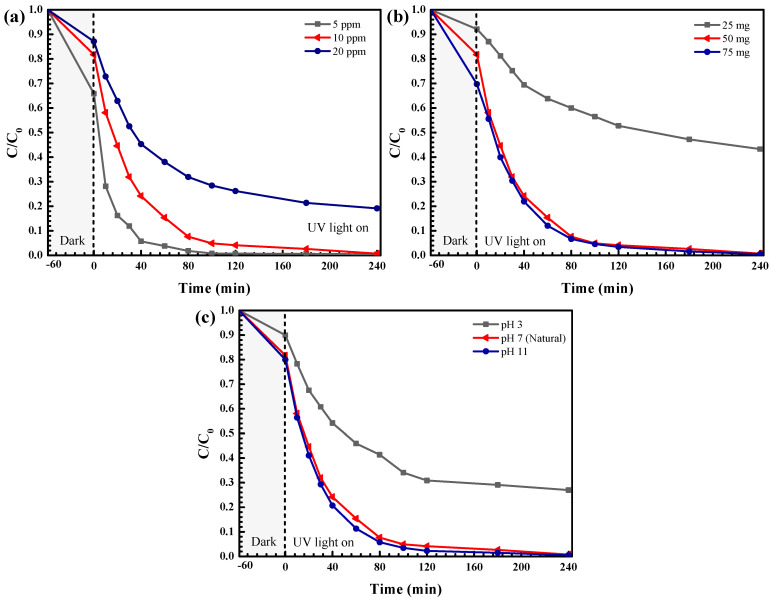
The effect of initial concentration (**a**), catalyst content (**b**), and initial solution pH (**c**) on the photodegradation of the OTC antibiotic.

The photodegradation of OTC and RR141 under sunlight was also investigated as seen in Figure 11. In terms of OTC removal, the efficiency reached 99% after 240 min, in the presence of the 5Ag-ZnO photocatalyst (Figure 10a). Accordingly, the rate constant of about 0.0545 min^−1^ was obtained (Figure 10c). The Ag-ZnO shows higher efficiency and a greater rate constant than the bare ZnO. In the case of RR141 removal, a similar trend was also detected. The silver decorated ZnO photocatalyst provided high performance of 100% after 240 min (Figure 10b). The rate constant of 0.0608 min^−1^ was detected. To sum up, the results from both UV light and solar light support the enhanced photoactivity of the silver decorated ZnO, compared to the pristine ZnO photocatalyst, due to the significant improvement of the charge separation at the interface [9,55].

#### 3.2.3. Effect of the Experimental Parameters on Photoactivity

The influence of some experimental parameters on OTC removal was elucidated. It can be seen from Figure 11a that enhancing the OTC concentration causes a decrease of degradation efficiency. The addition of OTC concentration results in the enhancement of light absorbed by the OTC molecules instead of the catalyst. Thus, the lowering of photon flux reaching the photocatalyst results in lowering of the photoactivity [18,19,20]. In the presence of 20 ppm OTC, the lowest photoactivity was detected. Even with the use of 10 ppm OTC, after 4 h of light irradiation, complete detoxification of the antibiotic was also achieved, so that the concentration of OTC was fixed at 10 ppm for further study.

The effect of catalyst loading on photoactivity was also investigated. As seen in Figure 11b, the addition of a catalyst causes an increase in the photoactivity due to the enhancement of OTC adsorption on the photocatalyst surface. [12]. However, the performance remains constant even addition of the catalyst, up to 75 mg. Therefore, a catalyst content of 50 mg was utilized for further investigation.

The effect of the initial solution pH (3–11) on photocatalytic activity was elucidated. The OTC aqueous solution showed a solution pH of about 7. The drastic decrease of the photoactivity at an extremely acidic condition (pH = 3) is mainly due to the dissolution of the photocatalyst [12]. It should be noted that high efficiency was still observed at the natural solution pH of about 7. There was no need to adjust the initial solution pH.

**Figure 12:**
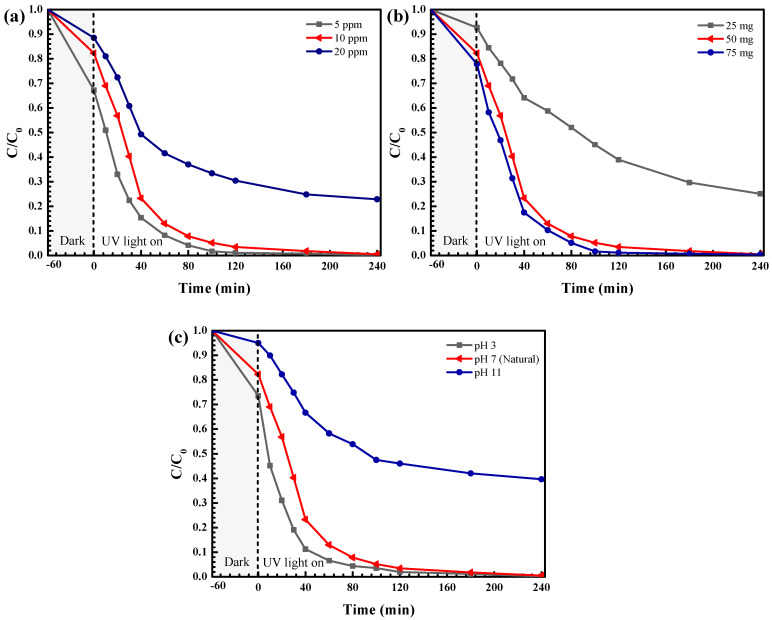
The effect of initial concentration (**a**), catalyst content (**b**), and initial solution pH (**c**) on photodegradation of RR141 dye.

Additionally, the effect of experimental parameters on RR141 degradation was also elucidated (Figure 12). As seen in Figure 12a, using a RR141 concentration of 5 ppm revealed the greatest photocatalytic performance. Lowering of the photoactivity with an increasing RR141 concentration was observed [2]. However, by fixing the RR141 concentration at 10 ppm, complete degradation of the dye can be obtained in 240 min. The effect of the catalyst loading on the photocatalytic performance was shown in Figure 12b. The addition of catalyst loading causes an enhancement of photoactivity. By using only 50 mg of the photocatalyst, a maximum photoactivity of 100% can be achieved within 240 min. The effect of pH on the photocatalytic efficiency was studied for pH values of 3–11 (Figure 12c). A dramatic drop in the performance at pH of 11 was mainly due to the repulsion between the anionic RR141 and the negative charge on the surface of ZnO (point of zero charge of about 10). The performance obtained from the natural pH of 7 is more or less as that obtained from the acidic condition (pH of 3). Therefore, there is no need to alter the initial solution pH of the azo dye [2].

#### 3.2.4. Photocatalytic Degradation Mechanism and Reusability

**Figure 13:**
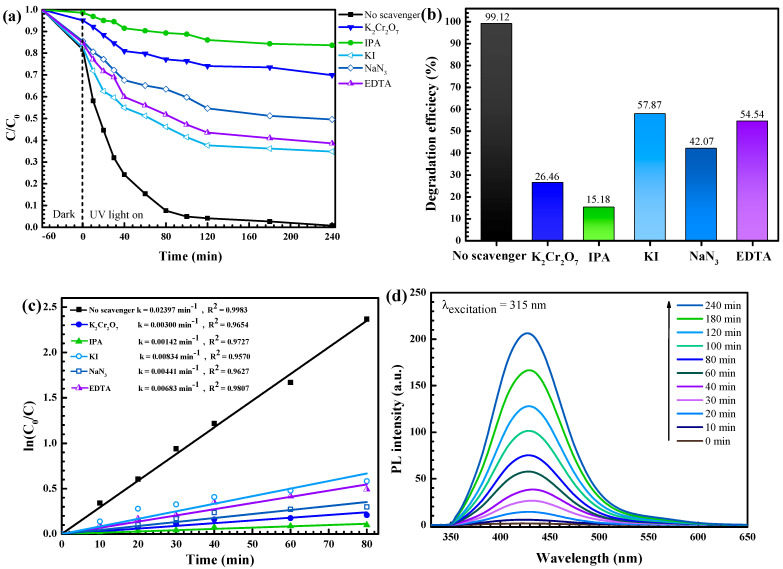
Lowering of C/C_0_ due to photodegradation (**a**), bar chart showing the photodegradation efficiency (**b**), the rate constants obtained from the degradation of OTC after the addition of various scavengers (**c**), and hydroxyl radical trapping PL spectra of the solution after photo illumination in the presence of the 5Ag-ZnO photocatalyst (**d**).

The mechanism based on photocatalytic degradation of the pollutant was determined from the trapping experiment [12,13]. The influence of some quenchers on the pollutant removal was elucidated. A dramatic decrease of photocatalytic performance was detected in the presence of IPA (Figure 13a,b) implying the crucial role of the hydroxyl radicals in the degradation of the antibiotic. The photogenerated electrons play a minor role as well.

Furthermore, confirmation of the photogenerated hydroxyl radical (•OH) was performed via the terephthalic acid (TA) probe method [12]. The fluorescence product, 2-hydroxyterephthalic acid (TA-OH), was observed. The addition of PL peak intensity at 425 nm with time (Figure 13d) indicates the important role of the hydroxyl radicals in the degradation of the toxic pollutant.

The important factors influencing the photocatalytic performance of the prepared catalyst are the crystallinity, the purity, and the morphology of the sample [7,9]. In theory, after light irradiation, the formation of electron-hole pairs were expected. Subsequently, the active species were created. All in all, the degradation mechanism of the harmful contaminant by the Ag-ZnO is proposed as follows.

Ag/ZnO + *hν* → Ag/ZnO + *e*^−^+ *h*^+^ (4)

*e*^−^ + O_2_ → •O_2_^−^ (5)

•O_2_^−^ + H^+^ → •HO_2_ (6)

*2e*^−^ + •HO_2_ + H^+^ → •OH + OH^−^ (7)

•O_2_^−^ + contaminant → products (8)

*e*^−^ + contaminant → products (9)

**Figure 14:**
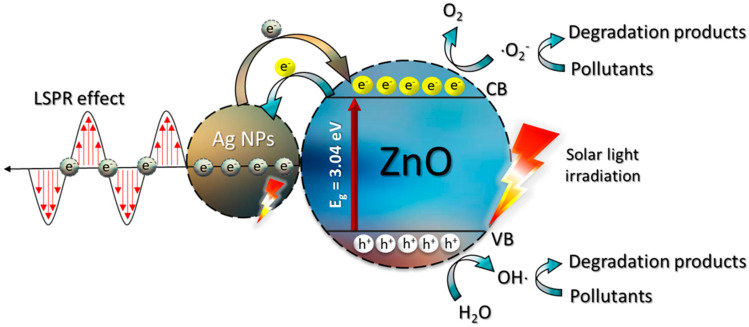
Photocatalytic mechanism schemes based on the degradation of the pollutants after photo irradiation of the 5Ag-ZnO photocatalyst.

Firstly, after photo illumination of the semiconductor, electrons were created in the conduction band (CB) while holes were generated in the valence band (VB). A reaction between photogenerated electrons and oxygen (O_2_) molecules will end up with the creation of superoxide anion radicals (•O_2_^−^). Finally, active species such as hydroxyl radicals (•OH) can be generated [7]. The pollutant was oxidized by the radicals to CO_2_, H_2_O, and some low molecular weight products [2,9,12]. The proposed degradation mechanism of the toxic contaminants by the prepared silver-ZnO is summarized in Figure 14.

The enhanced performance of the Ag-ZnO photocatalyst, compared to the bare photocatalyst, is attributed to the generation of the Schottky barrier between the interface of the decorated metal and the semiconductor [9]. Improvement of the quantum efficiency causes a dramatic increase in the charge carrier lifetime so that the promising photoactivity would be expected after well dispersion of metallic Ag on ZnO [55]. For better understanding, the pathway of RR141 degradation was studied previously in our group by using the LC-MS technique [2]. The mass spectrum obtained from the photodegradation intermediate products of RR141 dye was identified. The degradation mechanism was also proposed using the existence of some important breakdown products.

**Figure 15:**
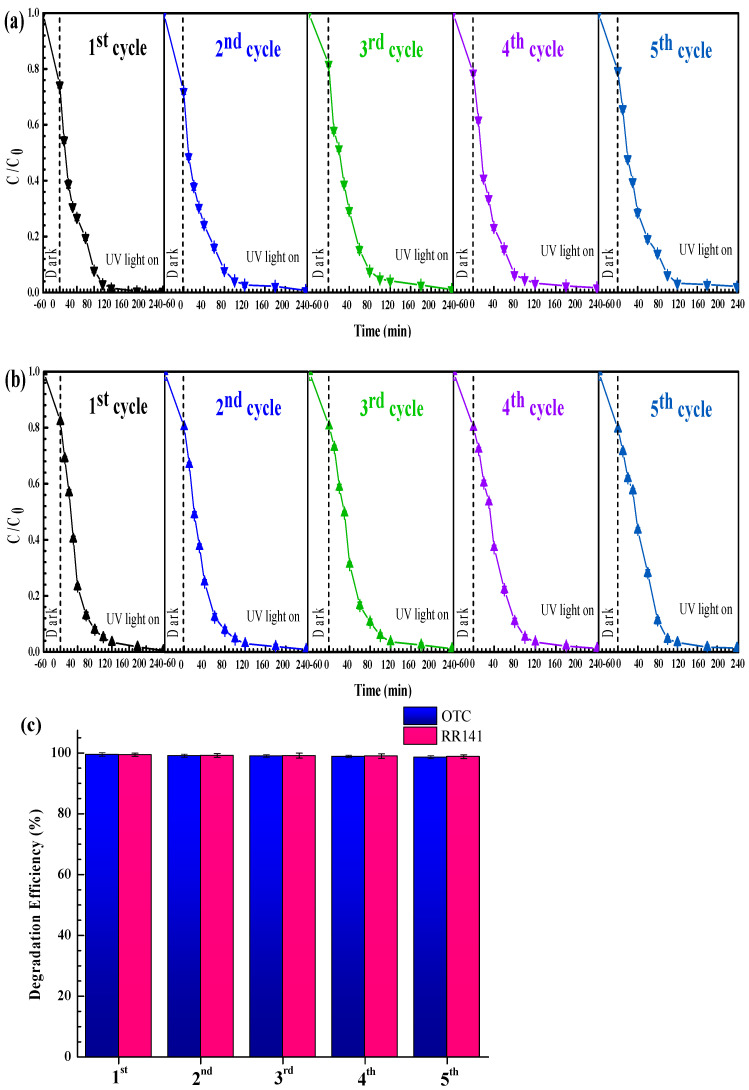
Reusability of the 5Ag-ZnO photocatalyst for photodegradation of OTC antibiotic (**a**) and RR141 dye (**b**), and the bar charts (**c**) showing cycling ability of the photocatalyst.

**Figure 16:**
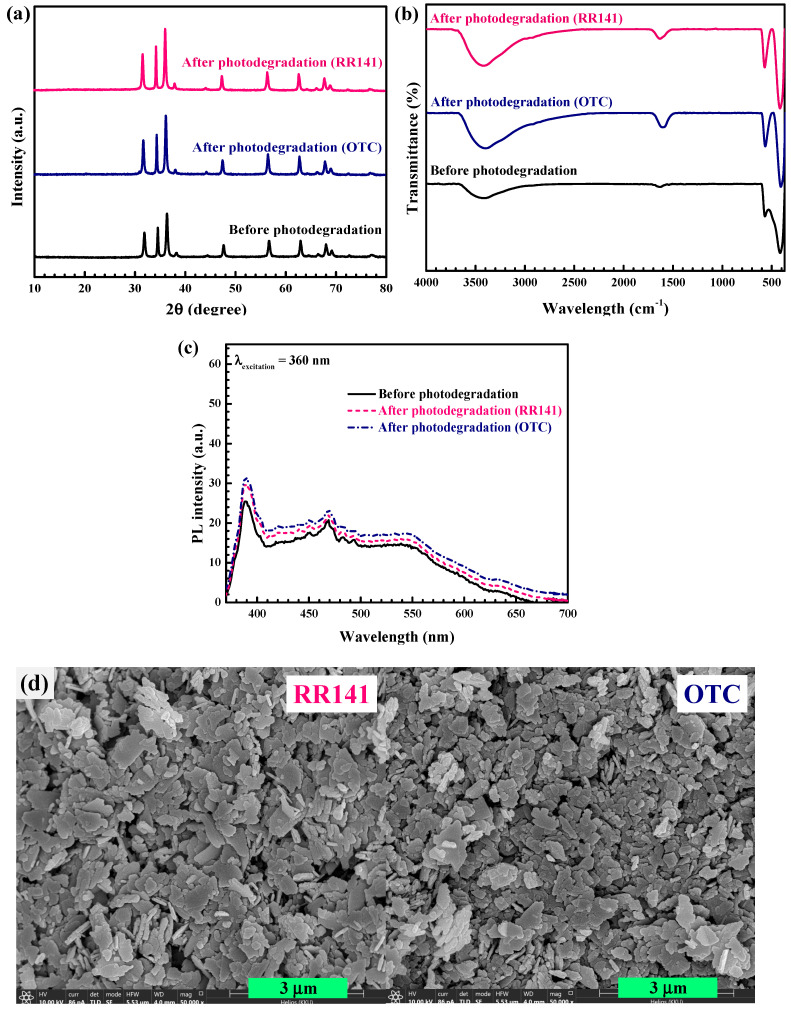
Comparison of the XRD patterns (**a**), vibrational spectra (**b**) and PL spectra (**c**) of the fresh and the used photocatalysts, and SEM images (**d**) of the 5Ag-ZnO photocatalyst after removal of RR141 dye and OTC antibiotics.

The cycling ability of the photocatalyst was also investigated [2,9,12,13]. Removal of OTC antibiotic and RR14 dye was examined for five successive runs. After the first round of photocatalytic study, the catalyst was separated, cleaned, and dried before use in the second round. The photoactivity after the fifth run (Figure 15) confirms the excellent cycling ability of the 5Ag-ZnO photocatalyst. In addition, the structural stability of the prepared catalyst was confirmed in Figure 16. The XRD patterns (Figure 16a), FT-IR spectra (Figure 16b), and PL spectra (Figure 16c) of the fresh and the used 5Ag-ZnO are similar. The SEM images from before and after the photodegradation study are also nearly the same (Figure 16d), implying the stability of the sample morphology [9]. As reported in the literatures, the improvement in the stability of the Ag- ZnO is mainly due to prolonging the charge carrier lifetime and increasing the charge separation efficiency at the heterojunction interface [9,55].

The removal of OTC and RR14 by various catalysts has been reported in the literatures [1,3,4,5,7,19,21,58,59,60,61,62,63,64,65,66,67,68,69]. In this work, the silver-ZnO was utilized to remove OTC and RR141. The photoactivity of 5Ag-ZnO together with those of other catalysts is shown in Table 1. On examining the RR141 removal, the bare ZnO showed 95% removal of the dye in 240 min [3]. In the case of SDS-capped and PVP-capped ZnO, a performance of 60–100% was achieved within 4 h [5,21]. The Zr-doped ZnO showed photoactivity of 96% after 40 min [58]. The ZnO/CdS photocatalyst showed high performance of 80% within 2 h [19]. The pristine CdS and Bi_4_MoO_9_ showed high performance of 68–100% after 4 h of irradiation [1,4,7]. In the present work, after 4 h, the silver-ZnO provided high photoactivity of 100% under UV light. In addition, after of only 2 h under sunlight, a promising performance of 99% was obtained. On examining OTC degradation, the bare BiVO_4_ exhibited a performance of 55–62% [12,13]. The pristine ZnO displayed 50% removal of OTC within 1.5 h [59]. By using the oxygen nanobubbles, a performance of 60% after 4 h was detected [60]. The binary composites based on TiO_2_ provided the photoactivity of 68–90% [61,62,63]. The graphitic carbon nitrile photocatalyst gave a performance of about 75% in 2 h [64]. Various binary and ternary heterojunctions provided the performance of about 70–97% [59,64,65,66,67,68,69]. Interestingly, in the present work, the silver-ZnO photocatalyst showed high performance of 99% within 4 h. All in all, the silver-ZnO in the present work exhibits high potential for detoxification of harmful OTC antibiotic and RR141 azo dye in natural water.

## 4. Conclusions

ZnO was first fabricated by a chemical precipitation method. After that, well dispersion of optimal silver metal on ZnO was demonstrated. The hexagonal 5Ag-ZnO catalyst (5 wt% Ag) exhibited a band gap energy of 3.00 eV. This photocatalyst showed the lowest PL intensity, compared to all samples including the bare ZnO. Accordingly, under sunlight, the highest photocatalytic performance of 100% toward degradation of OTC antibiotic and RR141 was obtained. The photodegradation of the pollutant follows the first-order reaction. The highest rate constant of 0.061 min^−1^ was reported. Hydroxyl radicals play the most important role in the degradation of the toxic pollutant. The photogenerated electrons also play a minor role in the removal of the contaminant. The prepared photocatalyst, with excellent stability, still shows high performance even after the fifth run, suggesting a promising cycling ability. The present finding demonstrates that the Ag-decorated ZnO photocatalyst is an excellent fit for environmental protection. The preparation of the photocatalyst film or magnetic separable photocatalyst is suggested for future work.

## Figures and Tables

**Table 1:** Comparison of RR141 azo dye and OTC antibiotic degradation using various photocatalysts.

Catalyst	Concentration	Catalyst Loading	Light Source	Lamp	Time (min)	Photodegradation (%)	Ref.
**● Photodegradation of RR141 azo dye**							
ZnO	10 mgL^−1^	50 mg	UV	125 W	240	95	[3]
SDS capped ZnO	10 mgL^−1^	50 mg	UV	125 W	240	100	[21]
SDS capped ZnO	10 mgL^−1^	50 mg	UV	125 W	240	95	[21]
SDS capped ZnO	10 mgL^−1^	50 mg	Visible	15 W	240	60	[21]
SDS capped ZnO	10 mgL^−1^	50 mg	Solar light	-	240	88	[21]
PVP capped ZnO	10 mgL^−1^	50 mg	UV	125 W	120	100	[5]
4% Zr(IV) doped ZnO	10 mgL^−1^	100 mg	UV	16 W	40	96	[58]
ZnO/CdS	10 mgL^−1^	50 mg	Visible	15 W	120	80	[19]
CdS	10 mgL^−1^	50 mg	Visible	15 W	240	95	[4]
CdS	10 mgL^−1^	50 mg	Visible	15 W	240	100	[1]
Bi_4_MoO_9_	10 mgL^−1^	50 mg	UV	125 W	240	68	[7]
5Ag-ZnO	10 mgL^−1^	50 mg	UV	125 W	240	100	This work
5Ag-ZnO	10 mgL^−1^	50 mg	Solar light	-	120	99	This work
**● Photodegradation of OTC antibiotic**							
BiVO_4_	10 mgL^−1^	50 mg	Visible	15 W	240	55.5	[13]
BiVO_4_	10 mgL^−1^	50 mg	Visible	15 W	240	62	[12]
ZnO	10 mgL^−1^	100 mg	Visible	300 W	150	50	[59]
Oxygen nanobubbles	30 mgL^−1^	-	UV	250 W	240	60	[60]
ZnO–TiO_2_	60 mgL^−1^	5 mg	Solar light	-	8	90.3	[61]
BiVO_4_/TiO_2_	10 mgL^−1^	-	Visible	1000 W	120	68.4	[62]
TiO_2_/RGO	50 mgL^−1^	60 mg	Natural light	-	60	80	[63]
Br-doped g-C_3_N_4_	10 mgL^−1^	250 mg	Visible	38.5 W	120	75	[64]
CdS QDs/LaMnO_3_	40 mgL^−1^	20 mg	Solar light	-	60	70	[65]
CoFe@NSC-1000	50 mgL^−1^	8 mg	Visible	300 W	150	90	[66]
ZnO/ZnFe_2_O_4_/diatomite	10 mgL^−1^	100 mg	Visible	300 W	150	95	[59]
NiCo/ZnO/g-C_3_N_4_	10 mgL^−1^	20 mg	Visible	300 W	50	71.3	[67]
Co_3_O_4_/TiO_2_/GO	10 mgL^−1^	25 mg	UV	300 W	90	91	[68]
Ag/AgCl/BiVO_4_	20 mgL^−1^	50 mg	UV	1000 W	120	97.6	[69]
5Ag-ZnO	10 mgL^−1^	50 mg	UV	125 W	240	99	This work
5Ag-ZnO	10 mgL^−1^	50 mg	Solar light	-	240	99	This work

## Data Availability

Not applicable.

## References

[1] Senasu T., Nanan S. (2017). Photocatalytic performance of CdS nanomaterials for photodegradation of organic azo dyes under artificial visible light and natural solar light irradiation. J. Mater. Sci. Mater. Electron..

[2] Kakarndee S., Nanan S. (2018). SDS capped and PVA capped ZnO nanostructures with high photocatalytic performance toward photodegradation of reactive red (RR141) azo dye. J. Environ. Chem. Eng..

[3] Chankhanittha T., Nanan S. (2018). Hydrothermal synthesis, characterization and enhanced photocatalytic performance of ZnO toward degradation of organic azo dye. Mater. Lett..

[4] Senasu T., Hemavibool K., Nanan S. (2018). Hydrothermally grown CdS nanoparticles for photodegradation of anionic azo dyes under UV-visible light irradiation. RSC Adv..

[5] Chankhanittha T., Watcharakitti J., Nanan S. (2019). PVP-assisted synthesis of rod-like ZnO photocatalyst for photodegradation of reactive red (RR141) and Congo red (CR) azo dyes. J. Mater. Sci. Mater. Electron..

[6] Mahmoodi N.M., Abdi J., Taghizadeh M., Taghizadeh A., Hayati B., Shekarchi A.A., Vossoughi M. (2019). Activated carbon/metal-organic framework nanocomposite: Preparation and photocatalytic dye degradation mathematical modeling from wastewater by least squares support vector machine. J. Environ. Manag..

[7] Chankhanittha T., Somaudon V., Watcharakitti J., Nanan S. (2021). Solar light-driven photocatalyst based on bismuth molybdate (Bi_4_MoO_9_) for detoxification of anionic azo dyes in wastewater. J. Mater. Sci. Mater. Electron..

[8] Chankhanittha T., Yenjai C., Nanan S. (2021). Utilization of formononetin and pinocembrin from stem extract of *Dalbergia parviflora* as capping agents for preparation of ZnO photocatalysts for degradation of RR141 azo dye and ofloxacin antibiotic. Catal. Today.

[9] Chankhanittha T., Komchoo N., Senasu T., Piriyanon J., Youngme S., Hemavibool K., Nanan S. (2021). Silver decorated ZnO photocatalyst for effective removal of reactive red azo dye and ofloxacin antibiotic under solar light irradiation. Colloids Surf. A Physicochem. Eng. Asp..

[10] Sansenya T., Masri N., Chankhanittha T., Senasu T., Piriyanon J., Mukdasai S., Nanan S. (2022). Hydrothermal synthesis of ZnO photocatalyst for detoxification of anionic azo dyes and antibiotic. J. Phys. Chem. Solids.

[11] Narenuch T., Senasu T., Chankhanittha T., Nanan S. (2021). Sunlight-Active BiOI Photocatalyst as an Efficient Adsorbent for the Removal of Organic Dyes and Antibiotics from Aqueous Solutions. Molecules.

[12] Hemavibool K., Sansenya T., Nanan S. (2022). Enhanced Photocatalytic Degradation of Tetracycline and Oxytetracycline Antibiotics by BiVO_4_ Photocatalyst under Visible Light and Solar Light Irradiation. Antibiotics.

[13] Senasu T., Youngme S., Hemavibool K., Nanan S. (2021). Sunlight-driven photodegradation of oxytetracycline antibiotic by BiVO_4_ photocatalyst. J. Solid State Chem..

[14] Yang X., Wang D. (2018). Photocatalysis: From Fundamental Principles to Materials and Applications. ACS Appl. Energy Mater..

[15] Chuenpratoom T., Hemavibool K., Rermthong K., Nanan S. (2021). Removal of Lead by Merlinoite Prepared from Sugarcane Bagasse Ash and Kaolin: Synthesis, Isotherm, Kinetic, and Thermodynamic Studies. Molecules.

[16] Wang H., Li Z., Yahyaoui S., Hanafy H., Seliem M.K., Bonilla-Petriciolet A., Dotto G.L., Sellaoui L., Li Q. (2021). Effective adsorption of dyes on an activated carbon prepared from carboxymethyl cellulose: Experiments, characterization and advanced modelling. Chem. Eng. J..

[17] Du X., Yang W., Liu Y., Zhang W., Wang Z., Nie J., Li G., Liang H. (2020). Removal of manganese, ferrous and antibiotics from groundwater simultaneously using peroxymonosulfate-assisted in-situ oxidation/coagulation integrated with ceramic membrane process. Sep. Purif. Technol..

[18] Chankhanittha T., Nanan S. (2021). Visible-light-driven photocatalytic degradation of ofloxacin (OFL) antibiotic and Rhodamine B (RhB) dye by solvothermally grown ZnO/Bi_2_MoO_6_ heterojunction. J. Colloid Interface Sci..

[19] Senasu T., Chankhanittha T., Hemavibool K., Nanan S. (2021). Visible-light-responsive photocatalyst based on ZnO/CdS nanocomposite for photodegradation of reactive red azo dye and ofloxacin antibiotic. Mater. Sci. Semicond. Process..

[20] Chankhanittha T., Somaudon V., Photiwat T., Youngme S., Hemavibool K., Nanan S. (2021). Enhanced photocatalytic performance of ZnO/Bi_2_WO_6_ heterojunctions toward photodegradation of fluoroquinolone-based antibiotics in wastewater. J. Phys. Chem. Solids.

[21] Juabrum S., Chankhanittha T., Nanan S. (2019). Hydrothermally grown SDS-capped ZnO photocatalyst for degradation of RR141 azo dye. Mater. Lett..

[22] Keshipour S., Mohammad-Alizadeh S. (2021). Nickel phthalocyanine@graphene oxide/TiO_2_ as an efficient degradation catalyst of formic acid toward hydrogen production. Sci. Rep..

[23] Bai Q., Shupyk I., Vauriot L., Majimel J., Labrugere C., Delville M.-H., Delville J.-P. (2021). Design of metal@titanium oxide nanoheterodimers by laser-driven photodeposition: Growth mechanism and modeling. ACS Nano.

[24] Bai Q., Lavenas M., Vauriot L., Le Tréquesser Q., Hao J., Weill F., Delville J.-P., Delville M.-H. (2019). Hydrothermal Transformation of Titanate Scrolled Nanosheets to Anatase over a Wide pH Range and Contribution of Triethanolamine and Oleic Acid to Control the Morphology. Inorg. Chem..

[25] Podasca V.E., Damaceanu M.D. (2021). ZnO-Ag based polymer composites as photocatalysts for highly efficient visible-light degradation of Methyl Orange. J. Photochem. Photobiol. A Chem..

[26] Wu F., Pu C., Zhang M., Liu B., Yang J. (2021). Silver embedded in defective twin brush-like ZnO for efficient and stable photocatalytic NO removal. Surf. Interfaces.

[27] Fiorenza R., Spitaleri L., Perricelli F., Nicotra G., Fragalà M.E., Scirè S., Gulino A. (2023). Efficient photocatalytic oxidation of VOCs using ZnO@Au nanoparticles. J. Photochem. Photobiol. A Chem..

[28] Nie M., Liao J., Cai H., Sun H., Xue Z., Guo P., Wu M. (2021). Photocatalytic property of silver enhanced Ag/ZnO composite catalyst. Chem. Phys. Lett..

[29] Chen J., Gu A., Miensah E.D., Liu Y., Wang P., Mao P., Gong C., Jiao Y., Chen K., Zhang Z. (2021). Silver-decorated ZIF-8 derived ZnO concave nanocubes for efficient photooxidation-adsorption of iodide anions: An in-depth experimental and theoretical investigation. J. Solid State Chem..

[30] Al-Mamun M.R., Islam M.S., Hossain M.R., Kader S., Islam M.S., Khan M.Z.H. (2021). A novel and highly efficient Ag and GO co-synthesized ZnO nano photocatalyst for methylene blue dye degradation under UV irradiation. Environ. Nanotechnol. Monit. Manag..

[31] Yudasari N., Anugrahwidya R., Tahir D., Suliyanti M.M., Herbani Y., Imawan C., Khalil M., Djuhana D. (2021). Enhanced photocatalytic degradation of rhodamine 6G (R6G) using ZnO–Ag nanoparticles synthesized by pulsed laser ablation in liquid (PLAL). J. Alloys Compd..

[32] Badán J.A., Jauregui G., Navarrete-Astorga E., Henríquez R., Jiménez F.M., Ariosa D., Dalchiele E.A. (2021). Solid-state thermal dewetted silver nanoparticles onto electrochemically grown self-standing vertically aligned ZnO nanorods for three-dimensional plasmonic nanostructures. Ceram. Int..

[33] Farooq M., Shujah S., Tahir K., Nazir S., Khan A.U., Almarhoon Z.M., Jevtovic V., Al-Shehri H.S., Hussain S.T., Ullah A. (2022). Ultra efficient 4-Nitrophenol reduction, dye degradation and Cr(VI) adsorption in the presence of phytochemical synthesized Ag/ZnO nanocomposite: A view towards sustainable chemistry. Inorg. Chem. Commun..

[34] Muñoz-Fernandez L., Gomez-Villalba L.S., Milošević O., Rabanal M.E. (2022). Influence of nanoscale defects on the improvement of photocatalytic activity of Ag/ZnO. Mater. Charact..

[35] Harinee S., Muthukumar K., James R.A., Arulmozhi M., Dahms H.U., Ashok M. (2022). Bio-approach ZnO/Ag nano-flowers: Enhanced photocatalytic and photoexcited anti-microbial activities towards pathogenic bacteria. Mater. Today Sustain..

[36] Lee Y., Fujimoto T., Yamanaka S. (2022). Characterization of submicro-sized Ag/ZnO particles generated using the spray pyrolysis method. Adv. Powder Technol..

[37] Liu H., Hu Y., Zhang Z., Liu X., Jia H., Xu B. (2015). Synthesis of spherical Ag/ZnO heterostructural composites with excellent photocatalytic activity under visible light and UV irradiation. Appl. Surf. Sci..

[38] Kaur A., Gupta G., Ibhadon A.O., Salunke D.B., Sinha A.S.K., Kansal S.K. (2018). A Facile synthesis of silver modified ZnO nanoplates for efficient removal of ofloxacin drug in aqueous phase under solar irradiation. J. Environ. Chem. Eng..

[39] Deng Q., Duan X., Ng D.H.L., Tang H., Yang Y., Kong M., Wu Z., Cai W., Wang G. (2012). Ag nanoparticle decorated nanoporous ZnO microrods and their enhanced photocatalytic activities. ACS Appl. Mater. Interfaces.

[40] Ansari S.A., Khan M.M., Lee J., Cho M.H. (2014). Highly visible light active Ag@ZnO nanocomposites synthesized by gel-combustion route. J. Ind. Eng. Chem..

[41] Liu H., Liu H., Yang J., Zhai H., Liu X., Jia H. (2019). Microwave-assisted one-pot synthesis of Ag decorated flower-like ZnO composites photocatalysts for dye degradation and NO removal. Ceram. Int..

[42] Yao Y., Zhang Y., Shen M., Li W., Xia W. (2020). The facile synthesis and enhanced photocatalytic properties of ZnO@ZnS modified with Ag0 via in-situ ion exchange. Colloids Surf. A Physicochem. Eng. Asp..

[43] Beura R., Pachaiappan R., Paramasivam T. (2021). Photocatalytic degradation studies of organic dyes over novel Ag-loaded ZnO-graphene hybrid nanocomposites. J. Phys. Chem. Solids.

[44] Ha L.P.P., Vinh T.H.T., Thuy N.T.B., Thi C.M., Van Viet P. (2021). Visible-light-driven photocatalysis of anisotropic silver nanoparticles decorated on ZnO nanorods: Synthesis and characterizations. J. Environ. Chem. Eng..

[45] Toe M.Z., Le A.T., Han S.S., Yaacob K.A.B., Pung S.Y. (2020). Silver nanoparticles coupled ZnO nanorods array prepared using photo-reduction method for localized surface plasmonic effect study. J. Cryst. Growth.

[46] Hassani A., Faraji M., Eghbali P. (2020). Facile fabrication of mpg-C3N4/Ag/ZnO nanowires/Zn photocatalyst plates for photodegradation of dye pollutant. J. Photochem. Photobiol. A Chem..

[47] Wang W., Zhang D., Ji Z., Shao D., Sun P., Duan J. (2021). High efficiency photocatalytic degradation of indoor formaldehyde with silver-doped ZnO/g-C3N4 composite catalyst under the synergistic effect of silver plasma effect and heterojunction. Opt. Mater..

[48] Iqbal S., Ahmad N., Javed M., Qamar M.A., Bahadur A., Ali S., Ahmad Z., Irfan R.M., Liu G., Akbar M.B. (2021). Designing highly potential photocatalytic comprising silver deposited ZnO NPs with sulfurized graphitic carbon nitride (Ag/ZnO/S-g-C3N4) ternary composite. J. Environ. Chem. Eng..

[49] Du C., Song J., Tan S., Yang L., Yu G., Chen H., Zhou L., Zhang Z., Zhang Y., Su Y. (2021). Facile synthesis of Z-scheme ZnO/Ag/Ag3PO4 composite photocatalysts with enhanced performance for the degradation of ciprofloxacin. Mater. Chem. Phys..

[50] Iqbal S., Bahadur A., Ali S., Ahmad Z., Javed M., Irfan R.M., Ahmad N., Qamar M.A., Liu G., Akbar M.B. (2021). Critical role of the heterojunction interface of silver decorated ZnO nanocomposite with sulfurized graphitic carbon nitride heterostructure materials for photocatalytic applications. J. Alloys Compd..

[51] Ye W., Jiang Y., Liu Q., Xu D., Zhang E., Cheng X., Wan Z., Liu C. (2022). The preparation of visible light-driven ZnO/Ag2MoO4/Ag nanocomposites with effective photocatalytic and antibacterial activity. J. Alloys Compd..

[52] Mohanty L., Pattanayak D.S., Dash S.K. (2021). An efficient ternary photocatalyst Ag/ZnO/g-C3N4 for degradation of RhB and MG under solar radiation. J. Indian Chem. Soc..

[53] Lucilha A.C., Camargo L.P., Liberatti V.R., Barbosa E.C.M., Dall’Antonia L.H. (2022). Zn1-xCoxO vs. Ag-ZnO photoanodes design via combustion: Characterization and application in photoelectrocatalysis. Colloids Surf. A Physicochem. Eng. Asp..

[54] Gea S., Situmorang S.A., Pasaribu N., Piliang A.F., Attaurrazaq B., Sari R.M., Pasaribu K.M., Goutianos S. (2022). Facile synthesis of ZnO–Ag nanocomposite supported by graphene oxide with stabilised band-gap and wider visible-light region for photocatalyst application. J. Mater. Res. Technol..

[55] Guo Y., Fu X., Xie Y., Zhu L., Liu R., Liu L. (2022). Synthesis of Ag/ZnO nanocomposites with enhanced visible photocatalytic performance. Opt. Mater..

[56] Singh S. (2022). Natural sunlight driven photocatalytic performance of Ag/ZnO nanocrystals. Mater. Today Commun..

[57] Kakarndee S., Juabrum S., Nanan S. (2016). Low temperature synthesis, characterization and photoluminescence study of plate-like ZnS. Mater. Lett..

[58] Christy E.J.S., Amalraj A., Rajeswari A., Pius A. (2021). Enhanced photocatalytic performance of Zr(IV) doped ZnO nanocomposite for the degradation efficiency of different azo dyes. Environ. Chem. Ecotoxicol..

[59] Xue L., Liang E., Wang J. (2022). Fabrication of magnetic ZnO/ZnFe2O4/diatomite composites: Improved photocatalytic efficiency under visible light irradiation. J. Mater. Sci. Mater. Electron..

[60] Wang L., Ali J., Wang Z., Oladoja N., Cheng R., Zhang C., Mailhot G., Pan G. (2020). Oxygen nanobubbles enhanced photodegradation of oxytetracycline under visible light: Synergistic effect and mechanism. Chem. Eng. J..

[61] Singh J., Kumar S., Rishikesh, Manna A.K., Soni R.K. (2020). Fabrication of ZnO–TiO2 nanohybrids for rapid sunlight driven photodegradation of textile dyes and antibiotic residue molecules. Opt. Mater..

[62] Wang W., Han Q., Zhu Z., Zhang L., Zhong S., Liu B. (2019). Enhanced photocatalytic degradation performance of organic contaminants by heterojunction photocatalyst BiVO4/TiO2/RGO and its compatibility on four different tetracycline antibiotics. Adv. Powder Technol..

[63] Zhen Q., Gao L., Sun C., Gong H., Hu P., Song S., Li R. (2018). Honeycomb-like TiO2@GO nanocomposites for the photodegradation of oxytetracycline. Mater. Lett..

[64] Hong J., Hwang D.K., Selvaraj R., Kim Y. (2019). Facile synthesis of Br-doped g-C3N4 nanosheets via one-step exfoliation using ammonium bromide for photodegradation of oxytetracycline antibiotics. J. Ind. Eng. Chem..

[65] Zhang H., Wang Y., Zhai C. (2022). Construction of a novel p-n heterojunction CdS QDs/LaMnO3 composite for photodegradation of oxytetracycline. Mater. Sci. Semicond. Process..

[66] Zhang S., Zhao S., Huang S., Hu B., Wang M., Zhang Z., He L., Du M. (2021). Photocatalytic degradation of oxytetracycline under visible light by nanohybrids of CoFe alloy nanoparticles and nitrogen-/sulfur-codoped mesoporous carbon. Chem. Eng. J..

[67] Wu J., Hu J., Qian H., Li J., Yang R., Qu L. (2022). NiCo/ZnO/g-C3N4 Z-scheme heterojunction nanoparticles with enhanced photocatalytic degradation oxytetracycline. Diam. Relat. Mater..

[68] Jo W.K., Kumar S., Isaacs M.A., Lee A.F., Karthikeyan S. (2017). Cobalt promoted TiO2/GO for the photocatalytic degradation of oxytetracycline and Congo Red. Appl. Catal. B Environ..

[69] Dai Y., Liu Y., Kong J., Yuan J., Sun C., Xian Q., Yang S., He H. (2019). High photocatalytic degradation efficiency of oxytetracycline hydrochloride over Ag/AgCl/BiVO4 plasmonic photocatalyst. Solid State Sci..

